# Taxonomic distribution and evolutionary analysis of the equol biosynthesis gene cluster

**DOI:** 10.1186/s12864-022-08426-7

**Published:** 2022-03-05

**Authors:** Keith Dufault-Thompson, Brantley Hall, Xiaofang Jiang

**Affiliations:** 1 National Library of Medicine, National Institutes of Health, Bethesda, Maryland USA; 2 Center for Bioinformatics and Computational Biology, University of Maryland, College Park, Maryland, USA

**Keywords:** Equol, Isoflavone, Human gut, Horizontal gene transfer, Equol-producing bacteria, *Eggerthellaceae*

## Abstract

**Background:**

Equol, an isoflavonoid metabolite with possible health benefits in humans, is known to be produced by some human gut bacteria. While the genes encoding the equol production pathway have been characterized in a few bacterial strains, a systematic analysis of the equol production pathway is currently lacking.

**Results:**

This study presents an analysis of the taxonomic distribution and evolutionary history of the gene cluster encoding the equol production pathway. A survey for equol gene clusters within the Genome Taxonomy Database bacterial genomes and human gut metagenomes resulted in the identification of a highly conserved gene cluster found in nine bacterial species from the *Eggerthellaceae* family. The identified gene clusters from human gut metagenomes revealed potential variations in the equol gene cluster organization and gene content within the equol-producing *Eggerthellaceae* clades. Subsequent analysis showed that in addition to the four genes directly involved in equol production, multiple other genes were consistently found in the equol gene clusters. These genes were predicted to encode a putative electron transport complex and hydrogenase maturase system, suggesting potential roles for them in the equol production pathway. Analysis of the gene clusters and a phylogenetic reconstruction of a putative NAD kinase gene provided evidence of the recent transfer of the equol gene cluster from a basal *Eggerthellaceae* species to *Slackia_A equolifaciens*, *Enteroscipio* sp000270285, and *Lactococcus garvieae* 20–92.

**Conclusions:**

This analysis demonstrates that the highly conserved equol gene cluster is taxonomically restricted to the *Eggerthellaceae* family of bacteria and provides evidence of the role of horizontal gene transfer in the evolutionary history of these genes. These results provide a foundation for future studies of equol production in the human gut and future efforts related to bioengineering and the use of equol-producing bacteria as probiotics.

**Supplementary Information:**

The online version contains supplementary material available at 10.1186/s12864-022-08426-7.

## Background

Equol is a highly estrogenic isoflavonoid compound produced through the activity of enteric bacteria and is thought to have positive effects on human health [1–3]. Equol is produced through the bacterial metabolism of the phytoestrogen daidzein, an isoflavone found in high concentrations in plants like soy [4, 5]. Equol has garnered particular interest due to its estrogenic activity [3] and potential health benefits, with isoflavonoid consumption and equol production having been associated with reduced prostate and breast cancer risk [6–9], lessening of menopause symptoms [10], and improved bone mineral density [11–13]. The importance of the gut microbiota in this process has been highlighted by the high inter-individual and population-level differences in equol production seen in human populations [14–16]. Despite the significant interest in equol production in the human gut, the study of the equol production pathway has been limited to only a few bacterial strains and the broader distribution of the genes encoding the pathway has not been extensively explored.

A majority of the previously identified equol-producing bacteria have been from the *Eggerthellaceae* family of the Actinobacteriota phylum, including *Adlercreutzia equolifaciens* [17], *Slackia_A isoflavoniconvertens* [18], and *Enteroscipio* sp000270285 (also known as *Eggerthella* sp. YY7918) [19]. The genetic basis of the equol production phenotype has been characterized in a few of these strains [20–25] and homologous genes encoding the equol production pathway have been subsequently identified in the genomes of other equol-producing bacteria [26, 27]. Outside of the *Eggerthellaceae* family, equol-producing strains have been identified in the *Bifidobacteriaceae* family, Gammaproteobacteria phylum, and Firmicutes phylum [4, 23, 28, 29]. The presence of this function in at least one Firmicutes strain, *Lactococcus garvieae* 20–92, was likely due to a horizontal gene transfer (HGT) event where the genes were acquired from an *Eggerthellaceae* species [21]. Horizontal gene transfer is the process by which genetic material is transferred from one organism to another and it often involves mobile genetic elements, which are regions of a genome that are able to be excised and transferred to new locations or other organisms [30]. HGT is an important part of bacterial evolution, providing a mechanism by which genes related to virulence, antibiotic resistance, and unique metabolic functions can be introduced into different bacterial clades [31]. One potential HGT event involving the transfer of the equol gene cluster has already been reported in a previous study [21], and the role of this process in the evolution and distribution of this gene cluster warrants further analysis.

The characterization of the equol production pathway has led to the identification of four genes directly involved in the conversion of daidzein to equol [20, 21, 25]. These genes encode four enzymes; daidzein reductase (DZNR), dihydrodaidzein racemase (DDRC), dihydrodaidzein reductase (DHDR), and tetrahydrodaidzein reductase (THDR), which catalyze the conversion of daidzein to equol through a series of four metabolic reactions [21, 23–25]. Additional characterization of this gene cluster has revealed that the expression of the genes is induced by daidzein and that genes are co-expressed [20, 21]. These previous studies of the equol gene cluster, however, have generally been focused on specific strains and typically only on the four genes directly involved in equol production. A systematic analysis of the gene cluster, its evolutionary history, and its distribution in other bacteria would provide a valuable foundation for the future study of equol metabolism. Additionally, the presence of other genes in the equol gene clusters has been noted in previous studies, but the degree to which these are conserved between species is poorly characterized, making it difficult to evaluate hypotheses about their potential roles in equol metabolism [20–22].

In this study, the taxonomic distribution and evolution of the equol gene cluster were investigated. A broad search for potential equol production gene clusters was performed in sequenced bacterial genomes and human gut metagenomes using gene sequences from known equol-producing bacteria. The search resulted in the identification of a highly conserved gene cluster found only in the *Eggerthellaceae* family of bacteria. The gene clusters typically included fifteen conserved genes consisting of the equol production genes, a putative electron transport flavoprotein complex, and a set of putative hydrogenase maturase enzymes. Further analysis of the gene synteny and sequence similarity between the gene clusters demonstrated that the gene cluster was likely gained through a horizontal gene transfer event in *Slackia_A equolifaciens* and *Enteroscipio* sp000270285. Furthermore, this study highlights the highly conserved nature of the equol gene cluster and provides a foundation for the future analysis of equol-producing bacteria.

## Results

### Taxonomic distribution of the equol production gene clusters

Putative equol production gene clusters were searched for in the 47,894 species representative prokaryotic genomes of the Genome Taxonomy Database (GTDB) (release 202). The sequences of the DZNR, DDRC, DHDR, and THDR genes from experimentally confirmed equol-producing bacteria were used as queries for this search (Supplemental Table 1). Putative equol gene clusters were identified in a total of 18 genomes from nine different bacterial species in the *Eggerthellaceae* family (Table 1, Supplemental Fig. 1). Ten of the genomes were from five species where at least one strain from that species had been previously identified as an equol producer (Supplemental Table 2), including four genomes of *A. equolifaciens*, two from *A. mucosicola*, two from *Slackia_A isoflavoniconvertens*, one from *Slackia_A equolifaciens,* and one from *Enteroscipio* sp000270285. The other eight genomes were from four additional species which have not been experimentally tested for equol production but can be considered putative equol-producing bacteria based on the presence of the equol gene cluster. These included *Senegalimassilia faecalis*, a species isolated from healthy human faeces [32], RUG013 sp001486445, a species represented by a metagenome-assembled genome (MAG) from a cow rumen metagenome [33], and CAG-1427 sp000435475 and CAG-1427 sp900556585, represented by MAGs assembled from human fecal metagenomes collected as part of the Metagenomics of the Human Intestinal Tract (MetaHIT) project. The equol gene clusters were all found in contiguous stretches of DNA, except for in one of the CAG-1427 sp900556585 genomes (GCF_900755045.1), where the THDR, DHDR, and conserved hypothetical protein genes were found on the end of a different contig from the rest of the gene cluster. The identified gene clusters were all from species belonging to the *Eggerthellaceae* family, showing that this gene cluster has an extremely limited taxonomic distribution.

**Table 1:** Presence of the equol gene cluster in GTDB genomes at the genus and species level

Genus	Species with Equol Gene Cluster / Total Species in Genus	Species with equol production gene clusters (genomes with equol gene cluster/total genomes for species)
***Adlercreutzia***	2/5	*A. equolifaciens* (4/5)
		*A. mucosicola* (2/2)
***Slackia_A***	2/5	*S. equolifaciens* (1/1)
		*S. isoflavoniconvertens* (2/8)
***Senegalimassilia***	1/3	*S. faecalis* (1/3)
***Enteroscipio***	1/2	*Enteroscipio* sp000270285 (1/1)
***CAG-1427***	2/20	*CAG-1427* sp900556585 (2/2)
		*CAG-1427* sp000435475 (1/3)
***RUG013***	1/1	*RUG013* sp001486445 (4/7)

**Fig. 1:**
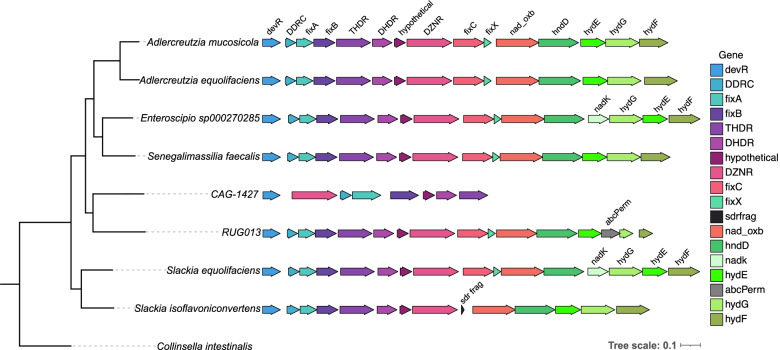
Equol Production Gene Cluster Organization. A diagram showing the organization of the detected equol production gene clusters organized based on their phylogenetic relationships. Phylogenetic relationships between the species based on a concatenated tree of conserved single-copy genes are shown on the left. Equol production gene clusters are shown on the right-hand side, with all gene clusters shown at the same scale. Only one representative gene cluster is shown for each of the species. The gene cluster shown for CAG-1427 is the gene cluster identified in CAG-1427 sp000435475 and represents the gene clusters of both CAG-1427 sp000435475 and CAG-1427 sp900556585

**Table 2:** Taxonomic assignments of detected metagenomic contigs with equol gene clusters

Genus	Species	Number of Contigs Detected
*Adlercreutzia*	*Adlercreutzia equolifaciens*	42
	*Adlercreutzia mucosicola*	2
	Unknown	7
*Slackia_A*	*Slackia_A equolifaciens*	6
	Unknown	1
*Senegalimassilia*	*Senegalimassilia faecalis*	1
*Unknown*	*Enteroscipio* sp000270285/*Slackia_A equolifaciens*	1

The presence of the gene clusters was found to be heterogeneous at the species and genus level (Table 1). The gene cluster was only found in two of the five *Adlercreutzia* species, being present in four out of five *A. equolifaciens* genomes and both of the *A. mucosicola* genomes. The genomic context of the equol gene cluster was found to be conserved in the one *A. equolifaciens* strain (genome GCF_009874275.1) that did not have the gene cluster (Supplemental Fig. 2A), corroborating the previously reported loss of the equol production genes in this strain [34]. The gene cluster was only found in one of three *Senegalimassilia faecalis* genomes, and the regions upstream and downstream of the gene cluster were found to be conserved in the other two genomes (Supplemental Fig. 2B). Similar variability was seen in the *Slackia_A* genus, where the gene cluster was detected in two of the eight *Slackia_A isoflavoniconvertens* genomes and in the one available *Slackia_A equolifaciens* genome but was not detected in any of the other species of the *Slackia_A* genus. The genomic context of the equol gene clusters in *S. isoflavoniconvertens* was found to be conserved in four of the other genomes, providing additional evidence that the gene cluster was missing from these genomes, but the remaining four genome assemblies were too fragmented to make any conclusions about these conserved regions (Supplemental Fig. 2C). The remaining gene clusters followed similar patterns of heterogeneity, typically being found in only one species from a genus (Table 1). The CAG-1427 sp000435475 and RUG013 sp001486445 did show variable presence of the gene cluster among the different genomes of the species, but the MAGs that make up these species are split into many contigs, making it difficult to analyze the genomic context of the equol genes. These results suggest that the equol gene cluster is not only taxonomically restricted to the *Eggerthellaceae* family but is also limited to only a few clades within that family.

**Fig. 2:**
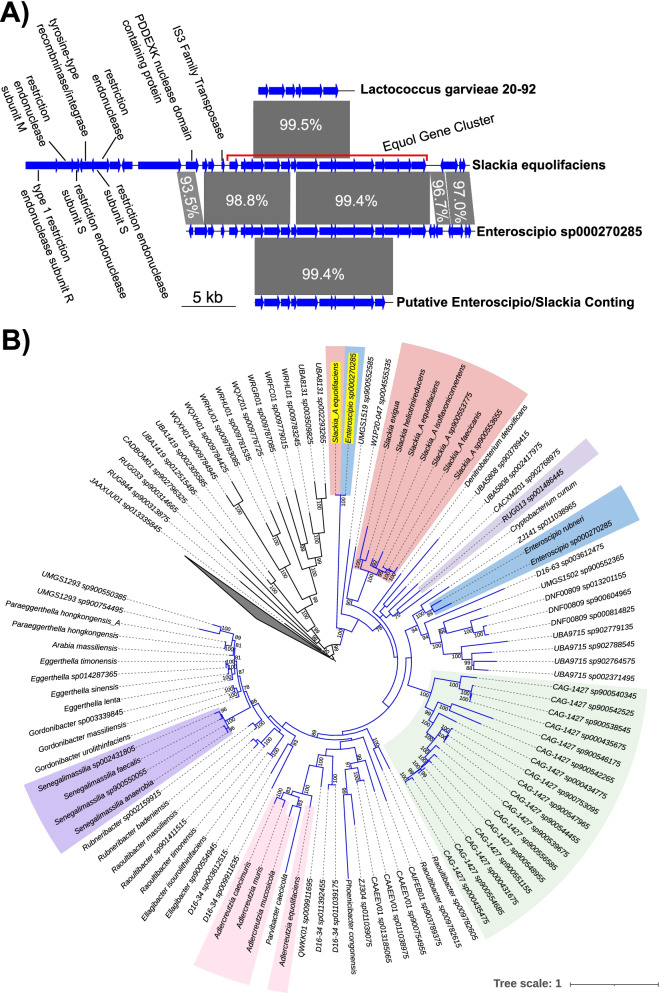
Potential HGT Region and *nadK* Phylogenetic Tree. **A**) Highly similar genomic regions containing the equol production gene cluster in *Slackia_A equolifaciens*, *Enteroscipio* sp000270285, and *L. garvieae* 20–92. Homologous regions between the nucleotide sequences are shown as shaded areas, with darker coloring meaning the genes had a high identity and the identity values being shown as the numbers in each shaded area. The region containing the equol gene cluster is highlighted with a red bar on the *Slackia_A equolifaciens* sequence. **B**) A phylogenetic tree based on the *nadK* homolog sequences identified in *Coriobacteriales* genomes. Genes from genera that are from species with identified equol gene clusters are colored by genus, and the *nadK* homologs from the *Slackia_A equolifaciens* and *Enteroscipio* sp000270285 equol gene clusters are highlighted with yellow backgrounds. Genes from the *Coriobacteriaceae, Atopobiaceae, and UMGS124* families were collapsed into a single leaf shown as a gray triangle. Branches leading to genes from the *Eggerthellaceae* family are colored blue. All bootstrap support values greater than 75% are shown

### Detection of equol production gene clusters in human gut metagenomes

Putative equol production gene clusters were identified in human gut metagenomes to find potential gene clusters from previously undetected strains. Out of 32,259 metagenomes samples from 114 projects, 60 contigs containing putative equol production gene clusters were identified from 18 of the projects (Supplemental Table 3). A majority of these contigs were assigned to the *Adlercreutzia* genus based on similarity to available genomes in GTDB. Forty-two contigs were assigned to the *A. equolifaciens* species, and two contigs were assigned to the *A. mucosicola* species (Table 2). An additional seven contigs were assigned to the *Adlercreutzia* genus but were not similar enough to be assigned to a specific species, potentially representing strains of *Adlercreutzia* without available genomes. The equol production gene clusters on the 42 *Adlercreutzia* contigs showed high similarity to the gene clusters detected in *A. equolifaciens* genomes, with the gene order being conserved in a majority of the contigs (Supplemental Fig. 3). The most notable difference seen in the gene clusters of the *Adlercreutzia* contigs was that the DZNR gene was not detected on eight of the contigs despite the surrounding genes being present.

Seven contigs were assigned to the *Slackia_A* genus, with six of them being assigned to the *Slackia_A isoflavoniconvertens* species and one of them being assigned only to the *Slackia_A* genus (Table 2). The gene clusters on the *Slackia_A* contigs had high similarity to the previously identified *Slackia_A isoflavoniconvertens* equol production gene clusters, with the gene order being conserved and the *fixCX* genes being missing from each of the contigs (Supplemental Fig. 4). One of the remaining contigs was assigned to the *Senegalimassilia faecalis* species and one was not able to be assigned to a specific species (Table 2). The gene cluster on the putative *Senegalimassilia faecalis* contig had high sequence similarity to the gene cluster from the *Senegalimassilia faecalis* genome. The last contig showed high similarity to the equol production gene clusters from the *Enteroscipio* sp000270285 and *Slackia_A equolifaciens* genomes but the contig only contained a portion of the gene cluster making it difficult to derive any further taxonomic assignment (Supplemental Fig. 5). Overall, the search for equol gene clusters in human gut metagenomes corroborated the limited taxonomic distribution of the equol gene cluster but suggested that there are likely additional uncharacterized strains of *Slackia_A* and *Adlercreutzia* that have this gene cluster.

### Equol gene cluster conserved genes and organization

The genomic context of the 18 detected equol production gene clusters was analyzed to identify additional conserved genes in the region (Fig. 1). In total, 18 different genes were identified within the putative equol production gene clusters, with eight of the genes being found in all the gene clusters and the rest being variably present. The four genes directly involved in equol production (DZNR, DDRC, DHDR, and THDR) were present in all 18 of the gene clusters. Additionally, a homolog of the DNA-binding transcriptional activator *devR* and a conserved hypothetical protein were also found in all the gene clusters. Homologs of the *fixABCX* genes which encode a putative electron bifurcating protein complex were also identified in the gene clusters, with the *fixA* and *fixB* genes being found in all 18 gene clusters and the *fixC* and *fixX* genes being found in 13 of the 18 gene clusters. Orthologs of a putative NADH oxidoreductase (*nad_oxb*), a putative NAD-reducing hydrogenase subunit (*hndD*), and three genes homologous to the Fe-hydrogenase maturase genes *hydE*, *hydG*, and *hydF* were also found in all the gene clusters except for those from the three CAG-1427 MAGs. This comparative analysis of the gene clusters highlights the degree to which this gene cluster is conserved among these different species.

### Identification of a possible HGT Event in *Slackia_A* and *Enteroscipio*

The gene cluster of *Slackia_A equolifaciens* was distinct from the clusters seen in the closely related *Slackia_A isoflavoniconvertens* species, being more similar in gene content and order to the gene cluster from *Enteroscipio* sp000270285 (Fig. 1). The gene clusters from these two species were the only ones that contained a putative NAD kinase gene (*nadK*) and the order of the hydrogenase maturase genes *hydGEF* was different in these two clusters compared to the other species (Fig. 1). A comparison of the genomic regions at the sequence level demonstrated the very high similarity (greater than 98% identity) between the *Slackia_A equolifaciens* and *Enteroscipio* sp000270285 gene clusters, with this same region being highly similar to the equol gene cluster fragment from *Lactococcus garvieae* 20–92 (Fig. 2A). Small, conserved regions upstream and downstream of the equol gene cluster were identified in *Slackia_A equolifaciens* and *Enteroscipio* sp000270285, which contained genes for a PD-(D/E)XK nuclease domain-containing protein and a putative IS3 family transposase. Additionally, genes encoding multiple subunits of a putative type 1 restriction endonuclease and a putative tyrosine type recombinase/integrase were identified in a non-conserved region just upstream of the equol gene cluster of *Slackia_A equolifaciens* (Fig. 2A). These features of the *Slackia_A equolifaciens* and *Enteroscipio* sp000270285 gene clusters and their high similarity to the gene cluster in the distantly related *L. garvieae* 20–92 strain, suggest that this variety of the equol gene cluster has been acquired through HGT events in these strains.

### Horizontal gene transfer of the equol gene cluster is reflected in the *nadK* gene phylogeny

The likely source of the horizontally transferred gene cluster in *Slackia_A equolifaciens*, *Enteroscipio* sp000270285, and *L. garvieae* 20–92 was investigated through a phylogenetic reconstruction of the *nadK* gene found in the equol gene clusters of *Slackia_A equolifaciens* and *Enteroscipio* sp000270285 (Fig. 2B). NAD kinase genes are thought to have evolved from a common ancestral NAD kinase and are typically found as single-copy genes in prokaryotes [35]. *nadK* genes were found in nearly all the *Eggerthellaceae* genomes as single-copy genes, but two *nadK* orthologs were found in *Slackia_A equolifaciens* and *Enteroscipio* sp000270285. A phylogenetic reconstruction of these *nadK* genes with other identified *nadK* homologs from the *Coriobacteriales* order showed that the genes from the equol gene clusters formed a distinct clade diverging from the rest of the *Eggerthellaceae nadK* genes early in the evolutionary history of the family. The distant relationship of these genes to the other *nadK* homologs found in the *Enteroscipio* sp000270285 and *Slackia_A equolifaciens* genomes suggests that these versions of the *nadK* gene did not originate from duplications in these organisms. Instead, it is likely that this version of the *nadK* gene originated as the result of an ancient duplication or movement into an equol gene cluster in an *Eggerthellaceae* species that is basal to the rest of the family. The similarity between the *nadK* genes and between the other genes present in the gene clusters suggests that the entire equol gene cluster was transferred from the unknown species, through a horizontal gene transfer event, to *Slackia_A equolifaciens*, *Enteroscipio* sp000270285, and *Lactococcus garvieae* 20–92.

## Discussion

Equol production by the gut microbiota has garnered much interest due to the likely health benefits of equol and the potential use of equol-producing bacteria as probiotics. The isolation and characterization of equol-producing bacteria from the human gut have led to the identification of multiple equol-producing strains of bacteria from the *Eggerthellaceae* family [21, 22, 36]. While the genes encoding the equol production pathway have been identified in some of these strains, a systematic search for the presence of this pathway in other organisms and a comparative analysis of the gene clusters from different taxa are lacking.

The search for putative equol production gene clusters resulted in the identification of only nine bacterial species from the *Eggerthellaceae* family that had the gene cluster. Most of these species have been identified as equol producers in previous studies (Supplemental Table 2), but the detection of the gene clusters in multiple strains that have not been reported to be equol-producers suggests that there are additional equol-producing species that have not yet been characterized. The extended analysis of putative equol gene clusters identified in human gut metagenomes provided further evidence of the extremely limited taxonomic scope of these genes, with nearly all the identified contigs having high similarity to the well-characterized equol producers from the *Slackia_A* and *Adlercreutzia* genera. This prevalence of *Slackia_A* and *Adlercreutzia* corroborates previous microbiome surveys, which have identified *A. equolifaciens* and *Slackia_A isoflavoniconvertens* strains as being significantly enriched in the guts of equol-producing humans [37, 38]. Although equol-producing bacteria have been identified in multiple taxa outside of the *Eggerthellaceae* family, including the *Bifidobacteria* [29, 39], *Lactobacillus* [4, 28], *Lactococcus* [23], and *Proteus* [40] genera, no plausible equol gene clusters were seen in these groups based on the initial survey or subsequent searches using more liberal thresholds. The lack of homologous genes in these other taxa suggests that equol production is carried out by different sets of enzymes in these bacteria and highlights the need for additional sequencing and experimental work to understand equol production in these groups.

The presence of the equol production gene cluster was found to be highly variable at the genus and species level. There have been previously reported strain-level differences in the presence of the equol production genes within the genus *Adlercreutzia*, with an apparent deletion of the equol production gene cluster being detected in *A. equolifaciens* W18.34a, *A. equolifaciens* MGYG-HGUT 02,480, and *A. celatus* AP38TSA [34]. The conserved presence of genes upstream and downstream of the gene cluster in *Senegalimassilia faecalis* and *Slackia_A isoflavoniconvertens* would also suggest that the gene cluster has either been lost in some strains or gained through horizontal gene transfer events in some clades. Gene loss events, like seen in *Adlercreutzia* [34], are a potential explanation for the variable presence of this gene cluster, especially if equol production provides little or no growth advantage to the bacteria as has been seen in some previous studies [20, 29, 41]. Horizontal gene transfer may also account for some of this variability, where the mobilization of the equol gene cluster may be responsible for the equol production genes being introduced into new clades.

Horizontal transfer of the genomic region containing the equol gene cluster has been suggested as the source of the equol genes in *L. garvieae* 20–92 [21]. The equol production genes in *L. garvieae* 20–92 were found to be highly similar to the corresponding genes from *Eggerthellaceae* species (greater than 99% identity to the genes from *Enteroscipio* sp000270285), despite *Lactococcus* being in a different phylum [36]. The genomic region containing the genes in *L. garvieae* 20–92 was also found to have a skewed GC content, a feature often used to detect DNA derived from foreign genomes, compared to what is typically seen in *Lactococcus* genomes [21]. Unfortunately, only a small region of DNA containing the equol genes was sequenced in *L. garvieae* 20–92, making it impossible to analyze the genomic region containing the genomes. The equol gene clusters in *Slackia_A equolifaciens* and *Enteroscipio* sp000270285 were found to be highly similar to the partial gene cluster sequenced from *L. garvieae* 20–92, suggesting a potential relationship between the gene clusters found in these three strains. The conserved synteny and high identity between the genes in the *Slackia_A equolifaciens*, *Enteroscipio* sp000270285, and *L. garvieae* 20–92 gene clusters, along with the subsequent phylogenetic analysis of the unique *nadK* gene provide evidence that these three strains have gained the entire equol gene cluster from a similar source through horizontal gene transfer events. Based on this evidence, there was likely a duplication or rearrangement event resulting in the formation of a *nadK*-containing equol gene cluster in a strain that is basal to the rest of the *Eggerthellaceae* family. This version of the equol gene cluster was eventually mobilized and transferred to *Slackia_A equolifaciens*, *Enteroscipio* sp000270285, and *L. garvieae* 20–92. The original source of the *nadK-*containing gene cluster is not yet known as no additional close homologs of the *nadK* genes were found in the other equol gene clusters. The additional presence of conserved genes encoding a putative IS3 family transposase and PD-(D/E)XK nuclease domain-containing protein in the conserved region of the *Slackia_A equolifaciens* and *Enteroscipio* sp000270285 gene clusters and the presence of genes commonly seen in mobile elements in the surrounding region of the *Slackia_A equolifaciens* gene cluster suggests that the region may be part of a mobile genetic element, providing a possible mechanism for the repeated transfer of the gene cluster to new strains.

The highly conserved nature of the equol gene cluster combined with previous evidence of the co-expression of genes within the cluster [21, 34] raises the possibility that the other genes found in the cluster have roles in equol production. The *fixABCX* genes encode a putative electron transport flavoprotein complex, with homologous genes being involved in the anaerobic metabolism of carnitine in *Escherichia coli* [42] and nitrogen in *Rhodospirillum rubrum* [43]. The overexpression of the *ydiS* gene, a putative oxidoreductase and distant homolog of *fixA*, in a transformed *E. coli* strain, dramatically increased the production of equol by the strain and allowed it to overcome the growth inhibition usually caused by equol in *E. coli* [44]. This evidence suggests that the *fixABCX* genes could play a role in equol production either through their involvement in the electron transfer associated with the conversion of daidzein to equol as has been previously suggested [21] or through another process like the maintenance of redox homeostasis.

While less evidence exists for the involvement of the remaining genes found in the gene cluster, their putative functions suggest possible roles that they may play in equol production. The *hydE* was previously seen to be co-expressed with the rest of the equol gene cluster and it was suggested that this gene might have a role in the maturation of the other enzymes in the gene cluster that contain Fe-S centers [20]. The results presented in this study show that two additional genes are conserved in most of the equol gene clusters, *hydF* and *hydG*, which together with *hydE* are known to be involved in the maturation of hydrogenase enzymes containing iron-sulfur centers [45, 46]. Based on the putative functions of these genes, it is possible that they play a role in the maturation of the other enzymes in the equol production pathway. The conserved hypothetical protein in the equol gene clusters has been predicted to have transmembrane helices [21] but specific functions for this gene are still unknown. The potential involvement of these other conserved genes in equol production could explain the low efficiency seen in some strains transformed with the equol production genes [44, 47] and the failure of some transformed strains to produce any equol [41], as these experiments focused on just the DZNR, DHDR, DDRC, and THDR enzymes.

This study provides context to the equol production phenotype demonstrating the restricted taxonomic nature of the gene cluster encoding this pathway and highlighting the potential involvement of additional genes in the production of equol. These observations have implications for the further use of equol-producing bacteria in the industrial production of equol and as probiotics. While HGT events may end up explaining the presence of the equol production phenotype in some additional strains, the lack of detected homologs of these genes in other taxa suggests that this would not explain the presence of the function in all cases. This highlights an important knowledge gap in terms of the genes responsible for this phenotype outside of the *Eggerthellaceae* family and demonstrates the need for further characterization of equol-producing strains from other taxa.

## Conclusions

The identification and subsequent analysis of equol production gene clusters revealed a highly conserved gene cluster only found within the *Eggerthellaceae* family of bacteria. The consistent presence of the *fixA*, *fixB*, and the conserved hypothetical protein genes in the equol production gene clusters, along with previous experimental evidence suggests that the function of these or similar genes may be important to the efficient utilization of the equol production pathway and has implications for future attempts at transforming this pathway into other bacterial strains. Analysis of the gene clusters provides evidence of the horizontal acquisition of this gene cluster in the *Enteroscipio* and *Slackia_A* genera suggesting a prominent role for HGT in the evolution of the equol production pathway.

## Methods

### Identification of putative equol production gene clusters

Genomes from 8 experimentally confirmed equol producing strains (Supplemental Table 1) were downloaded from GTDB release r202 [48, 49]. The protein sequences for the putative DZNR, DDRC, DHDR, and THDR genes were obtained from each of these genomes, aligned using MUSCLE version 3.8.31 [50], and used to generate HMM profiles of each gene using *hmmbuild* version 3.3.2 [51]. *hmmscan* version 3.3.2 [51] was used to search for putative orthologs in all the 47,894 bacterial and archaeal species cluster representative genomes in GTDB r202, and only hits with e-values less than 1e^−20^ were considered. Genomes were considered to have putative equol production gene clusters if at least three of the genes were detected in the same genomic neighborhood. For the purposes of this survey, a group of genes was considered to be in the same genomic neighborhood if the maximum distance between any two genes in the group was three genes or less. A search for putative orthologs of the equol production genes was performed on the 769 non-representative genomes within the Coriobacteriia class following the same procedure as the search against the GTDB representative genomes. Species with detected equol gene clusters were plotted onto the GTDB bacterial reference tree from GTDB version r202 [52] using iTOL [53].

### Organization of the equol production gene cluster

Orthologous protein groups within the 18 genomes with putative equol gene clusters were identified using Orthofinder version 2.5.2 [54] with default settings. The genes present in regions upstream and downstream of the equol production genes were compared across the 18 genomes to identify what genes were conserved in multiple genomes (Supplemental Table 1). A phylogenetic reconstruction of the 18 genomes was performed based on the conserved single-copy genes detected through the Orthofinder prediction. First, the sequences for 246 conserved single-copy genes were aligned using MUSCLE version 3.8.31 [50], and the alignments were concatenated. The concatenated alignment was trimmed using trimAl version 1.2 [55] to remove any positions where greater than 30% of the sequences had gaps. iQtree version 2.1.2 was used to perform a tree reconstruction using the best fit model found by iQtree (LG amino acid exchange rate matrix, empirical amino acid frequencies, with the FreeRate model of rate heterogeneity with five categories) with 1000 bootstraps [56]. One representative genome was chosen for each species and the equol gene clusters from the representative genomes were then visualized and plotted alongside the phylogenetic tree using the R packages *gggenes* and *ggtree* [57]. The conserved regions upstream and downstream of the equol gene clusters in *A. equolifaciens*, *S. faecalis*, and *Slackia_A isolflavoniconvertens* were visualized using clinker version 0.0.23 [58].

### Presence of equol production gene clusters in published metagenomes

Unassembled metagenomes were downloaded from 114 publicly available human gut metagenome sequencing projects. The 32,259 metagenomes were processed and assembled following a standardized protocol consisting of the following steps: 1) Reads were quality filtered and trimmed using TrimGalore version 0.6.6 with default settings. 2) The quality trimmed reads were mapped against the human genome version HG19 (GCF_000001405.13) using bowtie2 version 2.4.2 [59, 60] with default settings to detect possible contamination and the mapped reads were then removed using samtools version 1.11 [61]. 3) The trimmed and filtered metagenomes were then assembled using metaSPAdes version 3.14.1 [62] with default settings. The nucleotide sequences for the equol production genes from the 18 identified equol production gene clusters from the GTDB genomes, as well as the nucleotide sequences for the equol production genes from *Slackia_A* sp. NATTS and *L. garvieae* 20–92, were used as the query for a BLASTN search against the assembled metagenomic contigs. Only blast hits with greater than 30% identity, less than 1e^−5^ e-value, and greater than 70% coverage on the query sequence were considered quality hits. Metagenomic contigs with quality hits for at least three out of four of the equol production genes were used in subsequent analyses. Protein coding genes on the contigs were predicted using Prokka version 1.14.6 [63] with default settings. Contigs were assigned to different taxonomic groups at the species or genus level based on a BLASTn [64] search of the contig sequence against the GTDB representative genomes. All hits with identities greater than 90% and e-values less than 1e^−5^ were summarized to account for discontinuous hits. Summarized hits covering at least 40% of the contig were used for taxonomic assignment. Contigs having greater than 90% similarity to a genome were considered to be within the same genus, while contigs with greater than 95% similarity to a genome were considered to be the same species.

### Comparison of genomic regions and phylogenetic reconstruction of the *nadK* gene

A comparison of the genomic regions containing the equol gene clusters in *Slackia_A equolifaciens, Enteroscipio* sp000270285, and the partial gene cluster from *L. garvieae* 20–92 was performed using BLASTn to identify similar regions between the genomes [64]. Highly similar regions were identified based on the BLASTn results and were plotted using the genoPlotR package version 0.8.11in R [65]. A bi-directional best hit BLASTp approach was used to identify reciprocal best hits in the *Coriobacteriales* genomes from GTDB, for both copies of the *nadK* gene found in *Enteroscipio* sp000270285 and *S. equolifaciens*. Only hits with E-values less than 1e^−20^, identities greater than 30%, and query and target coverages greater than 70% were considered. The 505 *nadK* orthologs identified through the reciprocal best hit analysis, along with both pairs of *nadK* genes from *Slackia_A equolifaciens* and *Enteroscipio* sp000270285 were used to construct a multiple sequence alignment using Clustal Omega version 1.2.4 [66]. The multiple sequence alignment was trimmed using trimAl version 1.3 [55] to remove any positions where greater than 25% of the sequences had gaps. Identical sequences were then removed from the alignment so that only one copy was kept using the *dedup* function of Goalign version 0.3.5 [67]. In total, 182 out of 509 sequences were removed during the deduplication process. A tree was built using iQtree version 2.1.2 with 1000 ultrafast bootstraps using the best fit model detected by iQtree (LG amino acid exchange rate matrix, empirical amino acid frequencies, with the FreeRate model of rate heterogeneity with eight categories) [56, 68].

## Supplementary Information

**Additional file 1:** **Supplemental Fig. 1.** Presence of Equol Gene Clusters in Eggerthellaceae Genomes. A phylogenetic tree showing the presence of the equol gene cluster in the *Coriobacteriales *order. The *Coriobacteriaceae, Atopobiaceae*, and *UMGS124* families were collapsed into a single leaf shown as a gray triangle. Species where equol production gene clusters were detected are highlighted in yellow and the genera containing these species are represented by shaded areas and colored branches in the tree. **Supplemental Fig. 2.** Conserved regions upstream and downstream of the equol gene clusters. Gene synteny plots showing the conserved genes upstream and downstream of the equol gene clusters in **A) ***A. equolifaciens *, **B) ***S. faecalis*, and **C)** *Slackia_A isoflavoniconvertens*. Colored connections are shown between similar genes if the genes have greater than 75% amino acid identity. The equol gene clusters are highlighted with black boxes when present. Positions on the genome sequence are provided foreach of the visualized regions below the labels to the left of each plot. **Supplemental Fig. 3.** *Adlercreutzia *Contig Equol Gene Clusters. Equol production gene clusters from metagenomic contigs assigned to *A. equolifaciens*, *A. mucosicola*, or the *Adlercreutzia *genus. Gene clusters are shown at the same scale and detected gene clusters from published *A. equolifaceins *and *A. mucosicola *genomes are included for reference. **Supplemental Fig. 4.** *Slackia_A *Contig Equol Gene Clusters. Equol production gene clusters from metagenomic contigs assigned to *Slackia_A isoflavoniconvertens* or the *Slackia_A *genus. Gene clusters are shown at the same scale and detected gene clusters from published *Slackia_A.isoflavoniconvertens* and *Slackia_A equolifaciens *genomes are included for reference. **Supplemental Fig. 5.** *Senegalimassilia* and *Enteroscipio *Contig Equol Gene Clusters. Equol production gene clusters from metagenomic contigs assigned to *Senegalimassilia faecalis *and to *Enteroscipio* sp000270285 or *Slackia_A equolifaciens*. Gene clusters are shown at the same scale and detected gene clusters from published *Senegalimassilia faecalis *or *Enteroscipio* sp000270285 genomes are included for reference.

**Additional file 2: Supplemental Table 1.** GTDB genomes with detected equol gene clusters. **Supplemental Table 2.** Bacterial strains from the Coriobacteriales that have been reported to produce equol in previous studies. **Supplemental Table 3.** Metagenome Sequencing Projects with Detected Contigs Containing Putative Equol Gene Clusters.

## Data Availability

The datasets used and analyzed during this study are available in the GTDB repository (https://gtdb.ecogenomic.org/), Sequence Read Archive (https://www.ncbi.nlm.nih.gov/sra), and European Nucleotide Archive (https://www.ebi.ac.uk/ena/browser/home).

## Abbreviations

DZNR: Daidzein reductase
DHDR: Dihydrodaidzein reductase
DDRC: Dihydrodaidzein racemase
THDR: Tetrahydrodaidzein reductase
HGT: Horizontal gene transfer
MAG: Metagenome-assembled genome
