# Supplementary material for: Taxonomic distribution and evolutionary analysis of the equol biosynthesis gene cluster
Source: BMC Genomics. 2022 Mar 5;23:182. doi: 10.1186/s12864-022-08426-7 (PMC8898433; doi:10.1186/s12864-022-08426-7)
Supplement: Supplementary file 1 — Additional file 1: Supplemental Fig. 1. Presence of Equol Gene Clusters in Eggerthellaceae Genomes. A phylogenetic tree showing the presence of the equol gene cluster in the Coriobacteriales order. The Coriobacteriaceae, Atopobiaceae, and UMGS124 families were collapsed into a single leaf shown as a gray triangle. Species where equol production gene clusters were detected are highlighted in yellow and the genera containing these species are represented by shaded areas and colored branches in the tree. Supplemental Fig. 2. Conserved regions upstream and downstream of the equol gene clusters. Gene synteny plots showing the conserved genes upstream and downstream of the equol gene clusters in A) A. equolifaciens , B) S. faecalis, and C) Slackia_A isoflavoniconvertens. Colored connections are shown between similar genes if the genes have greater than 75% amino acid identity. The equol gene clusters are highlighted with black boxes when present. Positions on the genome sequence are provided foreach of the visualized regions below the labels to the left of each plot. Supplemental Fig. 3. Adlercreutzia Contig Equol Gene Clusters. Equol production gene clusters from metagenomic contigs assigned to A. equolifaciens, A. mucosicola, or the Adlercreutzia genus. Gene clusters are shown at the same scale and detected gene clusters from published A. equolifaceins and A. mucosicola genomes are included for reference. Supplemental Fig. 4. Slackia_A Contig Equol Gene Clusters. Equol production gene clusters from metagenomic contigs assigned to Slackia_A isoflavoniconvertens or the Slackia_A genus. Gene clusters are shown at the same scale and detected gene clusters from published Slackia_A.isoflavoniconvertens and Slackia_A equolifaciens genomes are included for reference. Supplemental Fig. 5. Senegalimassilia and Enteroscipio Contig Equol Gene Clusters. Equol production gene clusters from metagenomic contigs assigned to Senegalimassilia faecalis and to Enteroscipio sp000270285 or Sl [file 12864_2022_8426_MOESM1_ESM.zip › Supplemental-Figure-2 (1).pdf]

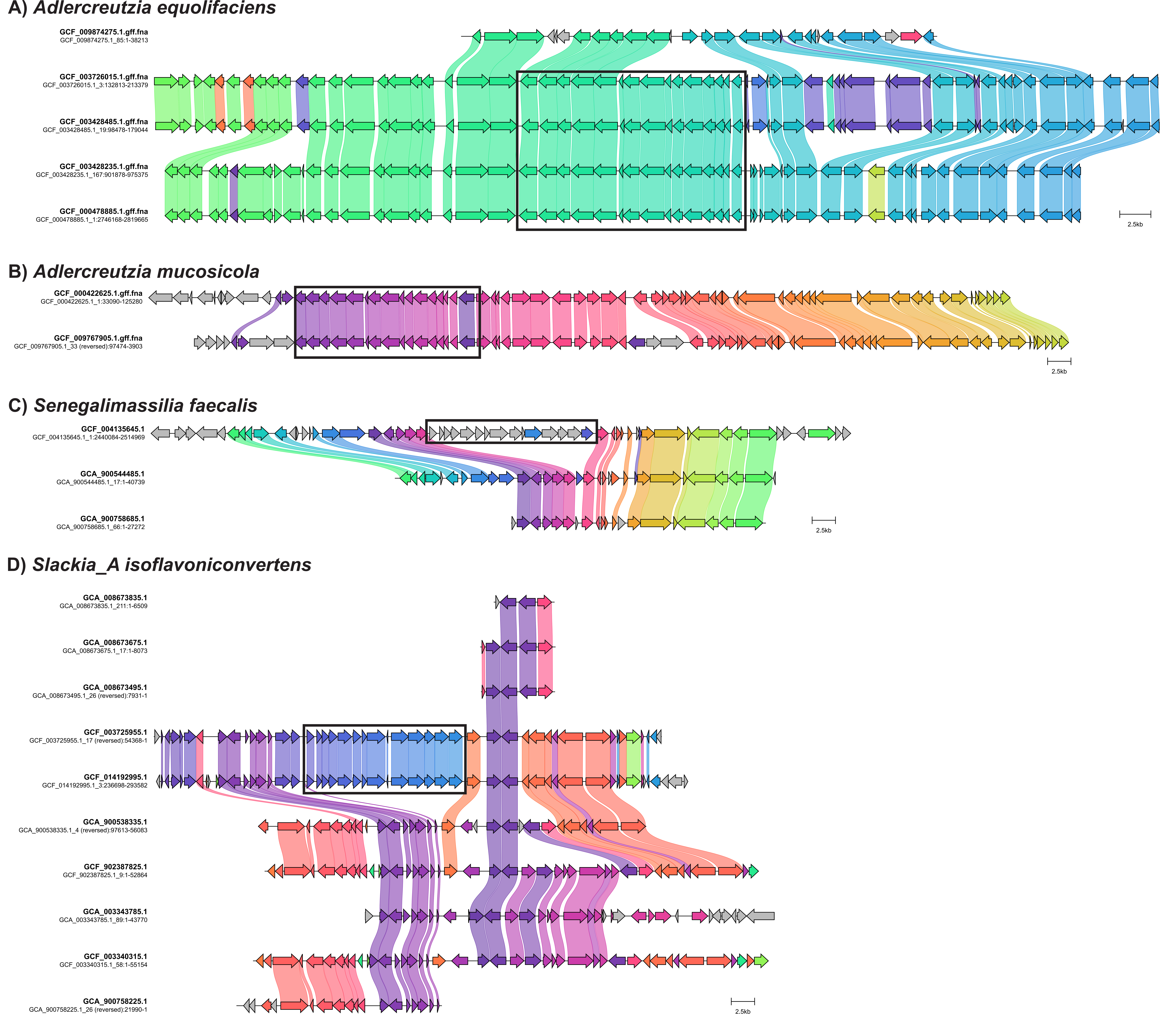

A) Adlercreutzia equolifaciens
GCF_009874275.1.gff.fna
GCF_009874275.1_85:1-38213
GCF_003726015.1.gff.fna
GCF_003726015.1_3:132813-213379
GCF_003428485.1.gff.fna
GCF_003428485.1_19:98478-179044
GCF_003428235.1.gff.fna
GCF_003428235.1_167:901878-975375
GCF_000478885.1.gff.fna
GCF_000478885.1_1:2746168-2819665
2.5kb
B) Adlercreutzia mucosicola
GCF_000422625.1.gff.fna
GCF_000422625.1_1:33090-125280
GCF_009767905.1.gff.fna
GCF_009767905.1_33 (reversed):97474-3903
2.5kb
C) Senegalimassilia faecalis
GCF_004135645.1
GCF_004135645.1_1:2440084-2514969
GCA_900544485.1
GCA_900544485.1_17:1-40739
GCA_900758685.1
GCA_900758685.1_66:1-27272
2.5kb
D) Slackia_A isoflavoniconvertens
GCA_008673835.1
GCA_008673835.1_211:1-6509
GCA_008673675.1
GCA_008673675.1_17:1-8073
GCA_008673495.1
GCA_008673495.1_26 (reversed):7931-1
GCF_003725955.1
GCF_003725955.1_17 (reversed):54368-1
GCF_014192995.1
GCF_014192995.1_3:236698-293582
GCA_900538335.1
GCA_900538335.1_4 (reversed):97613-56083
GCF_902387825.1
GCF_902387825.1_9:1-52864
GCA_003343785.1
GCA_003343785.1_89:1-43770
GCF_003340315.1
GCF_003340315.1_58:1-55154
GCA_900758225.1
GCA_900758225.1_26 (reversed):21990-1
2.5kb
