# Supplementary material for: Taxonomic distribution and evolutionary analysis of the equol biosynthesis gene cluster
Source: BMC Genomics. 2022 Mar 5;23:182. doi: 10.1186/s12864-022-08426-7 (PMC8898433; doi:10.1186/s12864-022-08426-7)
Supplement: Supplementary file 1 — Additional file 1: Supplemental Fig. 1. Presence of Equol Gene Clusters in Eggerthellaceae Genomes. A phylogenetic tree showing the presence of the equol gene cluster in the Coriobacteriales order. The Coriobacteriaceae, Atopobiaceae, and UMGS124 families were collapsed into a single leaf shown as a gray triangle. Species where equol production gene clusters were detected are highlighted in yellow and the genera containing these species are represented by shaded areas and colored branches in the tree. Supplemental Fig. 2. Conserved regions upstream and downstream of the equol gene clusters. Gene synteny plots showing the conserved genes upstream and downstream of the equol gene clusters in A) A. equolifaciens , B) S. faecalis, and C) Slackia_A isoflavoniconvertens. Colored connections are shown between similar genes if the genes have greater than 75% amino acid identity. The equol gene clusters are highlighted with black boxes when present. Positions on the genome sequence are provided foreach of the visualized regions below the labels to the left of each plot. Supplemental Fig. 3. Adlercreutzia Contig Equol Gene Clusters. Equol production gene clusters from metagenomic contigs assigned to A. equolifaciens, A. mucosicola, or the Adlercreutzia genus. Gene clusters are shown at the same scale and detected gene clusters from published A. equolifaceins and A. mucosicola genomes are included for reference. Supplemental Fig. 4. Slackia_A Contig Equol Gene Clusters. Equol production gene clusters from metagenomic contigs assigned to Slackia_A isoflavoniconvertens or the Slackia_A genus. Gene clusters are shown at the same scale and detected gene clusters from published Slackia_A.isoflavoniconvertens and Slackia_A equolifaciens genomes are included for reference. Supplemental Fig. 5. Senegalimassilia and Enteroscipio Contig Equol Gene Clusters. Equol production gene clusters from metagenomic contigs assigned to Senegalimassilia faecalis and to Enteroscipio sp000270285 or Sl [file Supplemental-Figure-3 (1).pdf › Supplemental-Figure-3 (1).pdf]

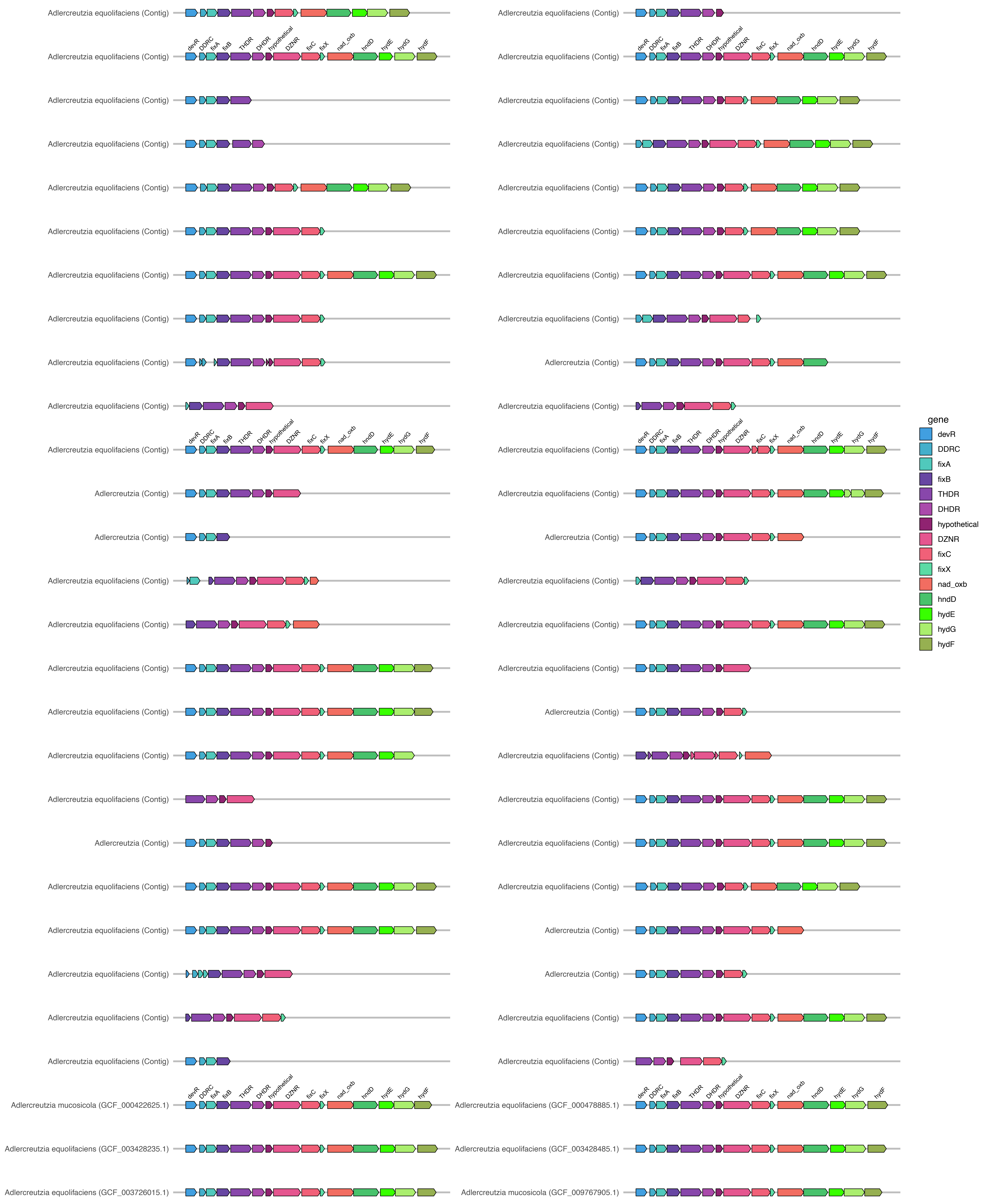
Adlercreutzia equolifaciens (Contig)
devR
DDRC
fixA
fixB
THDR
DHDR
hypothetical
DZNR
fixC
fixX
nad_oxb
hndD
hydE
hydG
hydF
Adlercreutzia (Contig)
Adlercreutzia mucosicola (GCF_000422625.1)
Adlercreutzia equolifaciens (GCF_003428235.1)
Adlercreutzia equolifaciens (GCF_003726015.1)
Adlercreutzia equolifaciens (GCF_000478885.1)
Adlercreutzia equolifaciens (GCF_003428485.1)
Adlercreutzia mucosicola (GCF_009767905.1)
gene
devR
DDRC
fixA
fixB
THDR
DHDR
hypothetical
DZNR
fixC
fixX
nad_oxb
hndD
hydE
hydG
hydF
