# Supplementary material for: Taxonomic distribution and evolutionary analysis of the equol biosynthesis gene cluster
Source: BMC Genomics. 2022 Mar 5;23:182. doi: 10.1186/s12864-022-08426-7 (PMC8898433; doi:10.1186/s12864-022-08426-7)
Supplement: Supplementary file 1 — Additional file 1: Supplemental Fig. 1. Presence of Equol Gene Clusters in Eggerthellaceae Genomes. A phylogenetic tree showing the presence of the equol gene cluster in the Coriobacteriales order. The Coriobacteriaceae, Atopobiaceae, and UMGS124 families were collapsed into a single leaf shown as a gray triangle. Species where equol production gene clusters were detected are highlighted in yellow and the genera containing these species are represented by shaded areas and colored branches in the tree. Supplemental Fig. 2. Conserved regions upstream and downstream of the equol gene clusters. Gene synteny plots showing the conserved genes upstream and downstream of the equol gene clusters in A) A. equolifaciens , B) S. faecalis, and C) Slackia_A isoflavoniconvertens. Colored connections are shown between similar genes if the genes have greater than 75% amino acid identity. The equol gene clusters are highlighted with black boxes when present. Positions on the genome sequence are provided foreach of the visualized regions below the labels to the left of each plot. Supplemental Fig. 3. Adlercreutzia Contig Equol Gene Clusters. Equol production gene clusters from metagenomic contigs assigned to A. equolifaciens, A. mucosicola, or the Adlercreutzia genus. Gene clusters are shown at the same scale and detected gene clusters from published A. equolifaceins and A. mucosicola genomes are included for reference. Supplemental Fig. 4. Slackia_A Contig Equol Gene Clusters. Equol production gene clusters from metagenomic contigs assigned to Slackia_A isoflavoniconvertens or the Slackia_A genus. Gene clusters are shown at the same scale and detected gene clusters from published Slackia_A.isoflavoniconvertens and Slackia_A equolifaciens genomes are included for reference. Supplemental Fig. 5. Senegalimassilia and Enteroscipio Contig Equol Gene Clusters. Equol production gene clusters from metagenomic contigs assigned to Senegalimassilia faecalis and to Enteroscipio sp000270285 or Sl [file Supplemental-Figure-1 (1).pdf › Supplemental-Figure-1 (1).pdf]

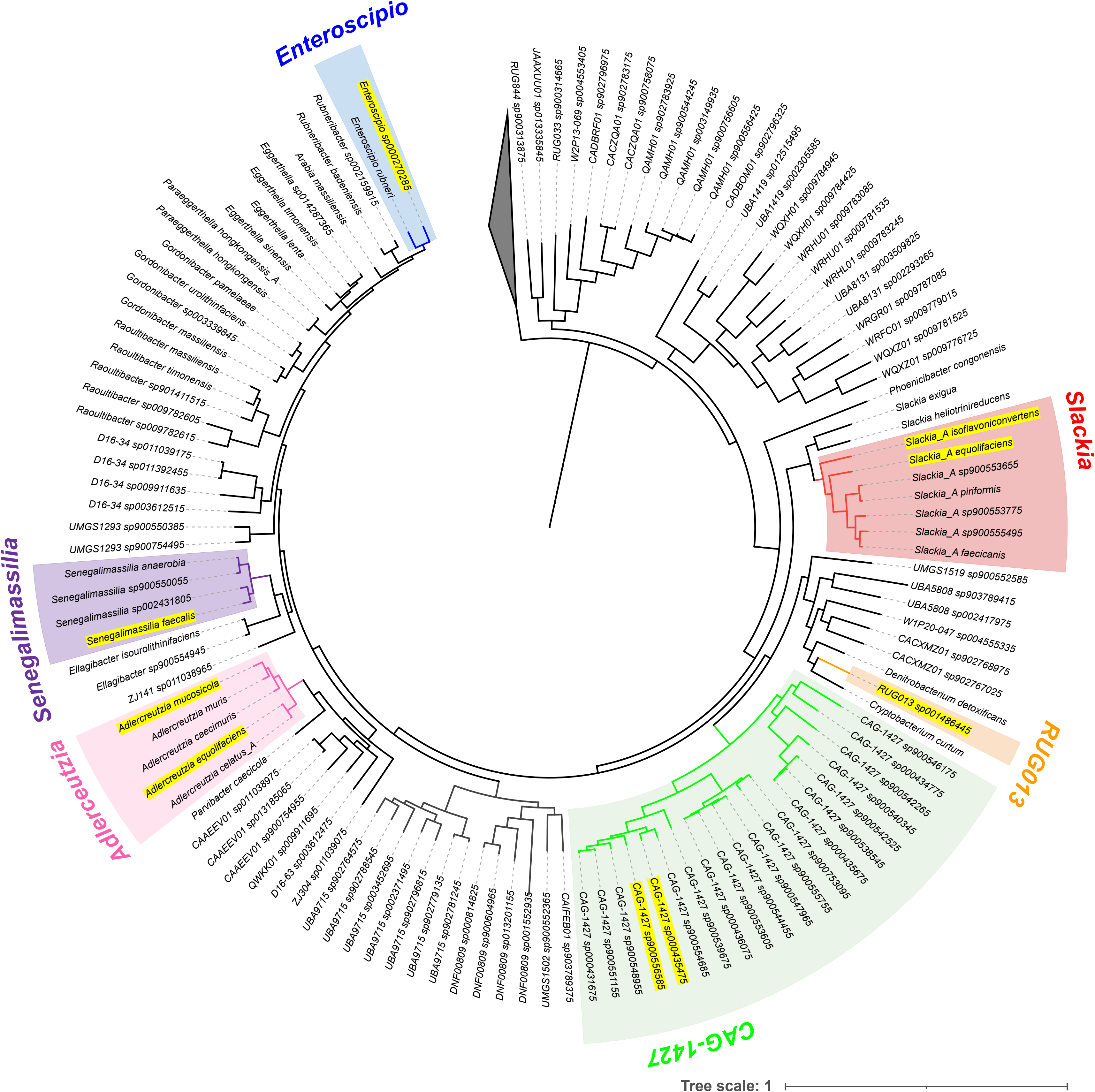

Enteroscipio
Slackia
RUG013
CAG-1427
Adlercreutzia
Senegalimassilia
RUG844 sp900313875
JAAXUU01 sp013335845
RUG033 sp900314665
W2P13-069 sp004553405
CADBRF01 sp902796975
CACZQA01 sp902783175
CACZQA01 sp900758075
QAMH01 sp902783925
QAMH01 sp900544245
QAMH01 sp003149935
QAMH01 sp900756605
QAMH01 sp900556425
CADBOM01 sp902796325
UBA1419 sp012515495
UBA1419 sp002305585
WQXH01 sp009784945
WQXH01 sp009784425
WRHU01 sp009783085
WRHU01 sp009781535
WRHL01 sp009783245
UBA8131 sp003509825
UBA8131 sp002293265
WRGR01 sp009787085
WRFC01 sp009779015
WQXZ01 sp009781525
WQXZ01 sp009776725
Phoenicibacter congonensis
Slackia exigua
Slackia heliotrinireducens
Slackia_A isoflavoniconvertens
Slackia_A equolifaciens
Slackia_A sp900553655
Slackia_A piriformis
Slackia_A sp900553775
Slackia_A sp900555495
Slackia_A faecicanis
UMGS1519 sp900552585
UBA5808 sp903789415
UBA5808 sp002417975
W1P20-047 sp004555335
CACXMZ01 sp902768975
CACXMZ01 sp902767025
Denitrobacterium detoxificans
RUG013 sp001486445
Cryptobacterium curtum
CAG-1427 sp900546175
CAG-1427 sp000434775
CAG-1427 sp900542265
CAG-1427 sp900540345
CAG-1427 sp900542525
CAG-1427 sp900538545
CAG-1427 sp000435675
CAG-1427 sp900753095
CAG-1427 sp900555755
CAG-1427 sp900547965
CAG-1427 sp900544455
CAG-1427 sp900553605
CAG-1427 sp000436075
CAG-1427 sp900539675
CAG-1427 sp900554685
CAG-1427 sp000435475
CAG-1427 sp900556585
CAG-1427 sp900548955
CAG-1427 sp900551155
CAG-1427 sp000431675
CAIFEB01 sp903789375
UMGS1502 sp900552365
DNF00809 sp001552935
DNF00809 sp013201155
DNF00809 sp900604965
DNF00809 sp000814825
UBA9715 sp902781245
UBA9715 sp902779135
UBA9715 sp902796815
UBA9715 sp002371495
UBA9715 sp003452695
UBA9715 sp902788545
UBA9715 sp902764575
ZJ304 sp011039075
D16-63 sp003612475
QWKK01 sp009911695
CAAEEV01 sp900754955
CAAEEV01 sp013185065
CAAEEV01 sp011038975
Parvibacter caecicola
Adlercreutzia celatus_A
Adlercreutzia equolifaciens
Adlercreutzia caecimuris
Adlercreutzia muris
Adlercreutzia mucosicola
ZJ141 sp011038965
Ellagibacter sp900554945
Ellagibacter isourolithinifaciens
Senegalimassilia faecalis
Senegalimassilia sp002431805
Senegalimassilia sp900550055
Senegalimassilia anaerobia
UMGS1293 sp900754495
UMGS1293 sp900550385
D16-34 sp003612515
D16-34 sp009911635
D16-34 sp011392455
D16-34 sp011039175
Raoultibacter sp009782615
Raoultibacter sp009782605
Raoultibacter sp901411515
Raoultibacter timonensis
Raoultibacter massiliensis
Gordonibacter massiliensis
Gordonibacter sp003339845
Gordonibacter urolithinfaciens
Gordonibacter pamelaeae
Paraeggerthella hongkongensis
Paraeggerthella hongkongensis_A
Eggerthella sinensis
Eggerthella lenta
Eggerthella timonensis
Eggerthella sp014287365
Arabia massiliensis
Rubneribacter badeniensis
Rubneribacter sp002159915
Enteroscipio rubneri
Enteroscipio sp000270285
Tree scale: 1
